# Supplementary material for: Research on the speed thresholds of trucks in a sharp turn based on dynamic rollover risk levels
Source: PLoS One. 2021 Aug 20;16(8):e0256301. doi: 10.1371/journal.pone.0256301 (PMC8378712; doi:10.1371/journal.pone.0256301)
Supplement: Attachment — Submitted filename: Response to Reviewers.docx [file pone.0256301.s001.docx]

Dear Editor Feng Chen and Reviewers,

Thanks very much for taking your time to review this manuscript. I really appreciate all your comments and suggestions! Please find my itemized responses in below and my revisions/corrections in the re-submitted files.

Thanks again!

**Response to the Suggestions from editor:**

1. Please ensure that your manuscript meets PLOS ONE's style requirements, including those for file naming.

**Response:** Thank you for the suggestion of file naming. We have made correction according the PLOS ONE style templates. Line4-8, the title of "Research on the Speed Thresholds of Trucks in a Sharp Turn Based on Dynamic Rollover Risk Levels" were corrected as "Research on the speed thresholds of trucks in a sharp turn based on dynamic rollover risk levels".

2. We note that one or more of the authors are employed by a commercial company: Shaoxing Communications Investment Group Co.,Ltd. and China State Construction Silkroad Construction Investment Group Co.,Ltd.

Please provide an amended Funding Statement declaring this commercial affiliation, as well as a statement regarding the Role of Funders in your study. If the funding organization did not play a role in the study design, data collection and analysis, decision to publish, or preparation of the manuscript and only provided financial support in the form of authors' salaries and/or research materials, please review your statements relating to the author contributions, and ensure you have specifically and accurately indicated the role(s) that these authors had in your study. You can update author roles in the Author Contributions section of the online submission form.

**Response:** Sorry for the trouble. Through verification, we found that Yafei Liu who is employed by China State Construction Silkroad Construction Investment Group Co., Ltd. played no role in the completion of the paper. After consultation with other authors, all authors consent to remove Yafei Liu from the author list. Line 11,16-18, the author list was corrected. We are very apologized for the inconvenience caused by the changes to authorship due to negligence. I have updated author roles in the Author Contributions section of the online submission form.

Funding Statement:

This research was supported by Scientific Research Project of Zhejiang Provincial Department of Transportation (No. 2020025) to JX and ZS. This project was completed by the cooperation between Chang'an University and Shaoxing Communications Investment Group Co., Ltd. The funder provided support in the form of salaries for authors, but did not have any additional role in study design, data collection and analysis, decision to publish, or preparation of the manuscript. The specific role of these author is articulated in the ‘author contributions’ section.

3.Please also provide an updated Competing Interests Statement declaring this commercial affiliation along with any other relevant declarations relating to employment, consultancy, patents, products in development, or marketed products, etc.

**Response:**

Competing Interests Statement:

There are no patents, products in development or marketed products association with this research to declare. This does not alter our adherence to PLOS ONE policies on sharing data and materials.

An updated Funding Statement and Competing Interests Statement are included in the cover letter.

4. We note you have included a table to which you do not refer in the text of your manuscript. Please ensure that you refer to Table 2 in your text; if accepted, production will need this reference to link the reader to the Table.

**Response:** Line411-413, the statement of "The rollover thresholds of the truck under three different loading conditions are calculated according to the theoretical model, which are shown in Table 2. " was added. Line414,the statement of "the table" was corrected as "table 2".

**Response to the reviewer’s comments:**

Reviewer #1:

1. The comparison of simulation test results and theoretical results are performed for radius of curve equals to 5500m, superelevation rate equals to 0%, longitudinal gradient equals to 0%, coefficient of friction 0.70 and speed equals to 100 km. How about the assessment for higher values of super elevation rate i.e., more than 0% in conjunction with speed exceeding 100km/h. Authors should perform more experiments with different values and compare those results with theoretical.

**Response:** According to the international standard, the minimum radius of the circular curve without setting superelevation under a design speed of 120km/h is 5500m, so we set a radius of 5500m and superelevation rate of 0%. In order to solve the reviewer’s doubt about whether simulation test results and theoretical results are consistent under the conditions of higher superelevation rate and higher speed, more experiments were supplemented. In the supplementary experiments, more superelevation and radius conditions based on design specification for highway alignment(JTG D20-2017) were added, which were superelevation rate equals to 2%( radius of curve equals to 3000m)、superelevation rate equals to 4% ( radius of curve equals to 2000m)、superelevation rate equals to 6%( radius of curve equals to 1000m), and longitudinal gradient is still 0%. Since the maximum speed of the selected typical truck is 105km/h, the speed in the supplementary experiments was set to 105km/h. The results of the supplementary experiments were shown in modified Table 3. The results of the supplementary experiments show simulation test results and theoretical results are still very consistent under the conditions of higher superelevation rate and higher speed. Correspondingly, Table 1 was also been modified, which added new scenes, and at this time, Line329-330, the statement of "Scenes 31-39 are used to test the rollover thresholds of the truck at a speed of 105km/h under different superelevation rates. "was added.

2. In line 514, again the authors concluded that the results are reliable and accurate and the scope of application is wide. However, I am still concerned about the limitation of different parameters. Why did the authors consider only flexible pavements (with friction coefficient equals to 0.70) and not rigid pavement?

**Response:** The supplementary experiments were added for verification. The results of the supplementary experiments show simulation test results and theoretical results are still very consistent under the conditions of higher superelevation rate and higher speed. The establishment of the road model requires the completion of road alignment modeling and pavement modeling with TruckSim, and pavement modeling only needs to input the road friction coefficient, so the software modeling cannot distinguish the performance difference between flexible roads and rigid roads. We study the road conditions in good weather. For new roads, under dry conditions, the road friction coefficient of asphalt concrete pavement and cement concrete pavement is 0.7~0.85. Therefore, it is set to 0.7 in the paper.

3. Change reference style and make it consistent.

**Response:** Thank you for your comments on the reference style. The style of all references was been carefully checked and revised.

Reviewer #2:

1. The reference style of this paper could be improved. For example, "Darren J. Torbic et al. [33]." could be "Torbic et al. [33]."

**Response:** Thank you for your comments on the reference style. The style of all references was been carefully checked and revised. Line 179, "Darren J. Torbic et al. [33]." was corrected as "Torbic et al. [33]."

2. More references about truck rollover risk are needed. For example, the following one.

[1] Reliability-based assessment of vehicle safety under adverse driving conditions, Transportation Research Part C: Emerging Technology, 18, 507-518.

**Response:** Line 545-546, the reference of "Reliability-based assessment of vehicle safety under adverse driving conditions, Transportation Research Part C: Emerging Technology, 18, 507-518. "was added.

3. "roll over" could be "rollover".

**Response:** Line97,127,129,240,257,268,402,467,the statement of "roll over" was corrected as "rollover".

The above is all the responses to the comments of the editor and reviewers. Thanks again to the editor and reviewers for your valuable suggestions and comments.

We hope that the revised manuscript is accepted for publication in the Journal of PLOS ONE.

Best regards! 

Sincerely,

Tian Xin
