# Supplementary material for: Research on the speed thresholds of trucks in a sharp turn based on dynamic rollover risk levels
Source: PLoS One. 2021 Aug 20;16(8):e0256301. doi: 10.1371/journal.pone.0256301 (PMC8378712; doi:10.1371/journal.pone.0256301)
Supplement: Attachment — Submitted filename: Response to Reviewers.docx [file pone.0256301.s002.docx]

Dear Editor Feng Chen and Reviewers,

Thanks very much for taking your time to review this manuscript. I really appreciate all your comments and suggestions! Please find my itemized responses in below and my revisions/corrections in the re-submitted files.

Thanks again!

**Response to the Suggestions from editor:**

1.Please review your reference list to ensure that it is complete and correct. If you have cited papers that have been retracted, please include the rationale for doing so in the manuscript text, or remove these references and replace them with relevant current references. Any changes to the reference list should be mentioned in the rebuttal letter that accompanies your revised manuscript. If you need to cite a retracted article, indicate the article’s retracted status in the References list and also include a citation and full reference for the retraction notice.

**Response:** Thank you for the suggestion of reference format. According to the PLOS ONE requirements. The format of each reference has been corrected and completed in line 523-680 of " Revised Manuscript with Track Changes". There is no retracted paper cited in the reference.

2. While revising your submission, please upload your figure files to the Preflight Analysis and Conversion Engine (PACE) digital diagnostic tool, <https://pacev2.apexcovantage.com/>. PACE helps ensure that figures meet PLOS requirements.

**Response:** We have uploaded the figure files to the PACE, and completed the figure files modification.

**Response to the reviewer’s comments:**

Reviewer #1:

1.The authors while responding have quoted " According to International Standards...", which they should avoid and explicitly mention the codes/standard. It’s better to revise the text accordingly.

**Response:** Thank you for the suggestion. The expression of "According to International Standards..." is not specific and should be avoid, which is revised as " According to AASHOTO,2018 and MOT, 2017".

where: AASHOTO,2018-American Association of State Highway and Transportation Officials. A Policy on Geometric Design of Highways and Streets. 7th Ed. Washington, D.C. 2018.

MOT, 2017- Ministry of transport of the People’s Republic of China (MOT). Design Specification for Highway Alignment, JTG D20-2017.1st ed. Beijing: China Communications Press; 2017.

The above is all the responses to the comments of the editor and reviewers. Thanks again to the editor and reviewers for your valuable suggestions and comments.

We hope that the revised manuscript is accepted for publication in the Journal of PLOS ONE.

Best regards! 

Sincerely,

Tian Xin
